# Exploratory study of the effectiveness of nebulised saline in children with neurodisability

**DOI:** 10.1183/13993003.01407-2020

**Published:** 2021-03-18

**Authors:** Natalia Galaz Souza, Andrew Bush, Hui-Leng Tan

**Affiliations:** 1 Imperial College London, London, UK; 2 Paediatrics, Royal Brompton and Harefield NHS Trust, London, UK

## Abstract

Respiratory morbidity is an important cause of hospitalisation and death in children with neurodisability [1]. Such children may have impaired respiratory function and inefficient cough due to weak bulbar and respiratory musculature, increased upper airway collapsibility and low lung compliance [2, 3]. Nebulised hypertonic saline (HS), usually 3% or 7%, is used to manage and prevent respiratory exacerbations in conditions such as cystic fibrosis (CF) and non-CF bronchiectasis. In patients with CF and non-CF bronchiectasis, nebulised HS has been associated with better airway clearance and lung function [4–8].

## Abstract 2

**The prescription of nebulised saline in children and young people with neurodisability was associated with improved respiratory outcomes and was favourably received by patients and their caregivers** https://bit.ly/3mSyzag

To the Editor:

Respiratory morbidity is an important cause of hospitalisation and death in children with neurodisability [1]. Such children may have impaired respiratory function and inefficient cough due to weak bulbar and respiratory musculature, increased upper airway collapsibility and low lung compliance [2, 3]. Nebulised hypertonic saline (HS), usually 3% or 7%, is used to manage and prevent respiratory exacerbations in conditions such as cystic fibrosis (CF) and non-CF bronchiectasis. In patients with CF and non-CF bronchiectasis, nebulised HS has been associated with better airway clearance and lung function [4–8]. In patients with acute bronchiolitis, comparisons of HS to isotonic saline (IS) have been inconclusive, with meta-analyses showing a reduction in length of hospital stay and risk of readmissions, although the bigger trials found no difference [9, 10]. The main mechanism of action proposed is an increase in the airway surface liquid volume and height in response to the osmotic gradient, hydrating secretions and facilitating their clearance [11]. Other mechanisms proposed include a reduction in mucus viscosity [12], thereby accelerating mucociliary clearance, and immunomodulatory [13, 14] and anti-microbial mechanisms [13, 15–17]. Nebulised saline is prescribed off-label to patients with neurodisability in concentrations ranging from 0.9% (IS) to 7% (HS), but there is no evidence of efficacy in this group [18]. We hypothesised that nebulised saline in children and young people with neurodisability would be associated with a decrease in respiratory exacerbations and antibiotic use, and improved ease of airway clearance. This is an observational study comparing outcomes during the first 12 months of the clinically determined prescription of nebulised saline with those of the preceding year. We explored patients' and parents' perception of the treatment.

This study was registered (ClinicalTrials.gov: NCT03623698) and approved by the regional research ethics committee (IRAS 243535, REC 18/YH/0103). Written informed consent was obtained from parents and, where possible, age- and developmentally appropriate assent from patients. Participants were recruited from a single London tertiary centre. Patients with neurodisability aged 1–18 years who had been prescribed nebulised saline (0.9%, 3%, 6% and/or 7%) to use daily or as required for at least 12 months, between 2011 and 2019, were included. These patients were prescribed nebulised saline because they had tenacious secretions and recurrent chest infections. Exclusion criteria were any additional respiratory comorbidity and lack of follow-up data.

All participants had an inhaled drug response assessment performed in the hospital before the medication was prescribed. This consisted of pre- and post-dose spirometry and peak cough flow (PCF), and monitoring of oxygen saturation and auscultation was also performed. If the child could not perform lung function testing, oxygen saturation and auscultation were used to determine bronchoconstriction. If they normally used inhaled bronchodilator before physiotherapy, then this was taken before the baseline lung function. If the patient failed the challenge, it was repeated at a later date giving an inhaled short-acting bronchodilator. The tonicity and regimen prescribed depended on the severity of the patient's respiratory morbidity, the availability of the preparation at their local health centre (7% *versus* 6%), and their response to the inhaled drug response assessment. This prescription was at the discretion of the medical team, independent of the study.

Data were collected retrospectively from medical records. Questionnaires were prospectively administered to patients and one parent for each child. As a quality control measure, because there was the possibility that hospital records were not complete, parents were asked about courses of oral and intravenous antibiotics and number of hospitalisations for respiratory infections in the year pre-treatment and during the first year where nebulised saline was prescribed, to check accuracy of hospital records. We assessed agreement between hospital records and parental reports using intraclass coefficient (ICC) and linear regression analysis.

Participants were asked to rate the ease of sputum clearance when they used and did not use nebulised saline. Patients used the “Facial Rating of Perceived Exertion Scale” [19] and parents used a 5-point Likert Scale. Patients aged 10–18 years and parents were asked to rate the usefulness of the treatment (very useful, useful, neither useful nor not useful, not useful, not useful at all). Open-ended questions were asked about what they found most useful, difficult or troublesome about the treatment.

Adjustments for confounding and demographic variables were performed with linear mixed-effects modelling with hospitalisations and courses of antibiotics as outcome measures, nebulised saline as fixed effect, while controlling for random effects, which were pre-defined from theory and literature. Pre-defined confounding variables were age, gender, scoliosis, bronchiectasis, being non-ambulant, antibiotic prophylaxis, use of a cough assist machine, home mechanical ventilation, tracheostomy, gastrostomy, dornase alpha nebulised treatment and scoliosis surgery. The Akaike Information Criterion was used to define the best model. Subgroup analyses were not performed because the sample size was small. Open questions were analysed using NVIVO and Topic Modelling analysis with Latent Dirichlet Allocation to identify hidden topics.

33 eligible patients were identified. Two patients refused consent, one was excluded because he also had CF, two because medical records were incomplete, and four did not attend the clinic appointments. Thus, 24 patients were included: nine patients had prescribed treatment with HS only (3–7%), four patients prescribed treatment with IS saline only, and 11 patients prescribed combinations of treatment with HS and IS. 17 patients had at least one saline tonicity prescribed for daily use, while eight patients used nebulised saline only as required. Only 1/24 patient was prescribed IS after a failed trial with HS. This patient experienced bronchospasm with 3% HS saline, and salbutamol produced tachycardia.

The mean age of children included was 11 years. Diagnoses included: spinal muscular atrophy (n=14), congenital myopathy (n=4), muscular dystrophies (n=3), Joubert syndrome, muscle eye brain disease, Rett syndrome (n=1 of each). Most patients were non-ambulant (88%), had scoliosis (54%), were gastrostomy fed (71%), and had home mechanical ventilation (67%). Only 3/24 patients had a tracheostomy. All patients used at least one form of airway clearance technique, including manual respiratory physiotherapy and mechanically assisted cough. Only 3/24 patients had a bronchodilator introduced to use pre-nebulisation with nebulised saline, of whom two had 7% saline and one had 3% saline. There was no significant difference in any respiratory treatment or medical procedure before and after the introduction of nebulised saline.

All patients who were able to cooperate and perform reliable lung function testing had spirometry and PCF assessments before (PCF: n=3, median 95 L·min^−1^, interquartile range (IQR) 73–107 L·min^−1^; forced vital capacity (FVC): n=8, median 26% pred, IQR 16–54% pred; forced expiratory volume in 1 s (FEV_1_): n=7, median 32% pred, IQR 20–58% pred) and after (PCF: n=5, median 85 L·min^−1^, IQR 70–95 L·min^−1^; FVC: n=5, median 28% pred, IQR 25–79% pred; FEV_1_: n=5, median 32% pred, IQR 23–84% pred) the prescription of nebulised saline.

The prescription of nebulised saline was associated with a decrease in the number of hospitalisations per year (before: median 1, IQR 0–2; *versus* after: median 0, IQR 0–0; 95% CI −1.5 to −0.5; p=0.001).

After adjustment for age, gender, use of home mechanical ventilation and antibiotic prophylaxis, the association continued to be strong (table 1). The adjusted estimated number of hospitalisations with prescription of nebulised saline was 0.38 compared to 1.40 in the preceding year. There was agreement between parental reports and hospital records (before: ICC=0.990; linear regression p-value, p=0.817; after: ICC=1.00).

**TABLE 1:** Mixed model analysis

**Parameters**	**Hospitalisations**				**Antibiotics**			
	**Estimate**	**p-value**	**95% CI**		**Estimate**	**p-value**	**95% CI**	
			**Lower bound**	**Upper bound**			**Lower bound**	**Upper bound**
**Estimates of fixed effects**								
Intercept	1.40	<0.001	0.84	1.96	8.38	0.05	−0.0003	16.77
Saline treatment	−1.02	<0.001	−1.53	−0.52	−2.88	0.001	−4.46	−1.29
**Estimates of covariance parameters**								
Intercept+random variables variance	0.001	0.583	<0.001	0.02	2.07	0.254	0.37	11.55
**Random effects parameters**	**Prediction**	**p-value**	**95% CI**		**Prediction**	**p-value**	**95% CI**	
			**Lower bound**	**Upper bound**			**Lower bound**	**Upper bound**
**Empirical best linear unbiased predictions**								
Intercept	<0.001	1.000	−1.50	1.50	<0.001	1.000	−5.01	5.01
Age	−0.02	0.150	−0.50	0.01	−0.13	0.157	−0.31	0.05
Male gender	−0.002	0.955	−1.23	1.23	−0.11	0.928	−3.47	3.26
Prophylactic antibiotic	−0.001	0.965	−1.12	1.19				
Home mechanical ventilation	−0.002	0.950	−1.25	1.25	−0.55	0.647	−3.91	2.81
Tracheostomised					1.31	0.319	−2.04	4.67
Gastrostomy					0.53	0.656	−2.80	3.87
Non-ambulant					−1.67	0.239	−5.12	1.75

Diagonal covariance structure was used for repeated measures and scaled identity covariance structure for random effects. Hospitalisations: adjusted for age, gender, prophylactic antibiotic and home mechanical ventilation. Antibiotics: adjusted for age, gender, home mechanical ventilation, having a tracheostomy, having a gastrostomy or being non-ambulant.

Nebulised saline was associated with a decrease in total courses of antibiotics for chest infections per year (before: median 4, IQR 2–6; *versus* after: median 1, IQR 0–2; 95% CI −4.0 to −1.5; p<0.001). This difference was also significant for oral antibiotics (before: median 2.5, IQR 1–6; *versus* after: median 1, IQR 0–2; 95% CI −3.0 to −1.0; p<0.001) and intravenous antibiotics (before: median 1, IQR 0–1; *versus* after: median 0, IQR 0–0; 95% CI −1.0 to 0.0; p=0.008). This association remained strong after adjusting for age, gender, use of home mechanical ventilation, tracheostomy, gastrostomy feeding and being non-ambulant. The estimated number of courses of antibiotics in the nebulised saline period was 5.5 compared to 8.4 in the preceding year. There was agreement between parental reports and hospital records for antibiotic use (intravenous: ICC=0.94, p=0.861; oral: ICC=0.98, p=0.513) and post-treatment (intravenous: ICC=0.93, p=0.484; oral: ICC=0.95, p=0.891).

All participants considered nebulised saline to be useful or very useful. Nebulised saline was associated with lower perceived effort during airway clearance reported by children (before: mean 6.45; *versus* after: mean 2.00; 95% CI −5,50 to −3,00; p=0.003) and parents (before: mean 4.21; *versus* after: mean 2.13; 95% CI −2,50 to −2,00; p<0.001). Most said that the treatment did not produce excess secretions (88% parents, 67% patients).

A common view was that nebulised saline helped “loosen secretions”, facilitating airway clearance and reducing the need to use suction. Some participants felt that it made the chest feel “less heavy” and that it helped unblock the nose and “alleviate sore throat”. Half of the parents and 25% of the children did not find anything complicated or troublesome about using nebulised saline. Concerns were expressed about wheeze (1/24 parents, 1/8 patients) and cough (1/8 patients), but these participants agreed that using a bronchodilator before nebulisation helped to prevent this. One parent reported perioral skin irritation. Two parents indicated that it was time-consuming, and four participants that the machine was noisy (one parent, three patients). One patient acknowledged that the mask strap was hard to remove for children with decreased mobility and strength, and one parent pointed out the difficulties in using the nebuliser for a child unable to sit because the device needed to be held in the vertical position.

Some limitations should be noted. This was a retrospective study, with potential for bias from self-selection. Possibly, participants who did not find the treatment useful discontinued it and consequently were excluded. The absence of a control group limits the study but using each patient as their own control to some extent mitigates this. To reduce the risk of confounding, a mixed model analysis was conducted to adjust for variables that could have influenced the results. The use of questionnaires to determine the accuracy of hospital records regarding number of hospitalisations and courses of antibiotics had the potential for recall bias. This study did not have adherence data. Another potential bias is the inclusion of patients who had a tracheostomy, as this may increase the amount of respiratory secretions, regardless of the diagnosis. However, the exclusion of these patients (n=3) from the study did not alter the main findings and the association with a decrease in number of hospitalisations, courses of antibiotics, and perceived effort in airway clearance remained strong. Finally, most patients used combinations of treatment with different concentrations of saline, so a dose effect could not be sought. Notwithstanding these issues, in the context of conditions in which either stability or at least some deterioration would be expected, the improvement in respiratory outcomes after the prescription of nebulised saline is striking.

We now need a randomised controlled trial of nebulised saline in children and young people with neuromuscular disorder to confirm benefit, determine the optimum regimens, and delineate mechanisms. This study provides data that will serve for future power calculations.

## Shareable PDF

10.1183/13993003.01407-2020.Shareable1 This one-page PDF can be shared freely online.
Shareable PDF ERJ-01407-2020.Shareable
