# Supplementary material for: Exploratory study of the effectiveness of nebulised saline in children with neurodisability
Source: Eur Respir J. 2021 Mar 18;57(3):2001407. doi: 10.1183/13993003.01407-2020 (PMC7970020; doi:10.1183/13993003.01407-2020)
Supplement: Supplementary file 1 [file ERJ-01407-2020.Shareable.pdf]

SHAREABLE PDF

Exploratory study of the effectiveness of nebulised saline in children with neurodisability

Natalia Galaz Souza[1], Andrew Bush[1,2] and Hui-Leng Tan[1,2]

Affiliations: [1]Imperial College London, London, UK. [2]Paediatrics, Royal Brompton and Harefield NHS Trust, London, UK.

Correspondence: Natalia Galaz Souza, 106 Ropewalk Court, Nottingham, NG1 5AD, UK.
E-mail: natalia.galaz-souza16@imperial.ac.uk

@ERSpublications
The prescription of nebulised saline in children and young people with neurodisability was associated with improved respiratory outcomes and was favourably received by patients and their caregivers https://bit.ly/3mSyzag

Cite this article as: Galaz Souza N, Bush A, Tan H-L. Exploratory study of the effectiveness of nebulised saline in children with neurodisability. *Eur Respir J* 2021; 57: 2001407 [https://doi.org/10.1183/13993003.01407-2020].

This single-page version can be shared freely online.

To the Editor:

Respiratory morbidity is an important cause of hospitalisation and death in children with neurodisability [1]. Such children may have impaired respiratory function and inefficient cough due to weak bulbar and respiratory musculature, increased upper airway collapsibility and low lung compliance [2, 3]. Nebulised hypertonic saline (HS), usually 3% or 7%, is used to manage and prevent respiratory exacerbations in conditions such as cystic fibrosis (CF) and non-CF bronchiectasis. In patients with CF and non-CF bronchiectasis, nebulised HS has been associated with better airway clearance and lung function [4–8]. In patients with acute bronchiolitis, comparisons of HS to isotonic saline (IS) have been inconclusive, with meta-analyses showing a reduction in length of hospital stay and risk of readmissions, although the bigger trials found no difference [9, 10]. The main mechanism of action proposed is an increase in the airway surface liquid volume and height in response to the osmotic gradient, hydrating secretions and facilitating their clearance [11]. Other mechanisms proposed include a reduction in mucus viscosity [12], thereby accelerating mucociliary clearance, and immunomodulatory [13, 14] and anti-microbial mechanisms [13, 15–17]. Nebulised saline is prescribed off-label to patients with neurodisability in concentrations ranging from 0.9% (IS) to 7% (HS), but there is no evidence of efficacy in this group [18]. We hypothesised that nebulised saline in children and young people with neurodisability would be associated with a decrease in respiratory exacerbations and antibiotic use, and improved ease of airway clearance. This is an observational study comparing outcomes during the first 12 months of the clinically determined prescription of nebulised saline with those of the preceding year. We explored patients' and parents' perception of the treatment.

Copyright ©ERS 2021.. This version is distributed under the terms of the Creative Commons Attribution Licence 4.0.
